# 4-Aminobenzoic Acid-Coated Maghemite Nanoparticles as Potential Anticancer Drug Magnetic Carriers: A Case Study on Highly Cytotoxic *Cisplatin*-Like Complexes Involving 7-Azaindoles

**DOI:** 10.3390/molecules19021622

**Published:** 2014-01-28

**Authors:** Pavel Štarha, Martin Stavárek, Jiří Tuček, Zdeněk Trávníček

**Affiliations:** 1 Regional Centre of Advanced Technologies and Materials, Department of Inorganic Chemistry, Faculty of Science, Palacký University, 17. listopadu 12, Olomouc CZ 77146, Czech Republic; E-Mails: pavel.starha@upol.cz (P.Š.); martin.stavarek01@upol.cz (M.S.); 2 Regional Centre of Advanced Technologies and Materials, Department of Experimental Physics, Faculty of Science, Palacký University, 17. listopadu 12, Olomouc CZ 77146, Czech Republic; E-Mail: jiri.tucek@upol.cz

**Keywords:** maghemite, nanoparticles, magnetic, platinum complexes, 7-azaindole derivatives, drug delivery

## Abstract

This study describes a one-pot synthesis of superparamagnetic maghemite-based 4-aminobenzoic acid-coated spherical core-shell nanoparticles (PABA@FeNPs) as suitable nanocomposites potentially usable as magnetic carriers for drug delivery. The PABA@FeNPs system was subsequently functionalized by the activated species (1* and 2*) of highly *in vitro* cytotoxic *cis*-[PtCl_2_(*3Cl*aza)_2_] (**1**; *3Cl*aza stands for 3-chloro-7-azaindole) or *cis*-[PtCl_2_(*5Br*aza)_2_] (**2**; *5Br*aza stands for 5-bromo-7-azaindole), which were prepared by a silver(I) ion assisted dechlorination of the parent dichlorido complexes. The products 1*@PABA@FeNPs and 2*@PABA@FeNPs, as well as an intermediate PABA@FeNPs, were characterized by a combination of various techniques, such as Mössbauer, FTIR and EDS spectroscopy, thermal analysis, SEM and TEM. The results showed that the products consist of well-dispersed maghemite-based nanoparticles of 13 nm average size that represent an easily obtainable system for delivery of highly cytotoxic *cisplatin*-like complexes in oncological practice.

## 1. Introduction

The platinum(II) complexes, such as cisplatin, oxaliplatin or carboplatin, are well-established anticancer chemotherapeutics used worldwide for the treatment of various types of cancer [1]. Application of these drugs causes several negative side-effects, such as nephrotoxicity, neurotoxicity or myelosuppression, which represent a permanent incentive for bioinorganic chemists to find novel non-platinum drugs (e.g., ruthenium complexes) or platinum complexes with diminished side-effects or to study the possibilities of the targeted drug delivery of the known as well as novel cytotoxic platinum complexes to the tumour tissues. The latter option, *i.e.*, targeted drug delivery, offers various approaches, as reviewed e.g., in [2,3,4]. One of them is based on the use of magnetic nanoparticles (NPs) coated with a suitable shell, comprising e.g., organic molecules of the same entity, that is able to interact with the drug [5,6]. A distribution of such systems within the organism suffering with cancer could be affected by an external magnetic field, whose application concentrates the drug into the tumour tissue. 

Among all the magnetic nanoparticles of transition metals and their oxides, iron oxide-based nanosystems hold a paramount position in various medical fields due to their promising properties including magnetic (e.g., superparamagnetism, strong magnetic response and saturation under small applied magnetic fields, excellent heating performance in the frequencies of the alternating magnetic field safe for humans) and biochemical (e.g., very low toxicity, biocompatibility, biodegradability) features [7,8]. To date, they have been successfully employed as negative contrast agents in magnetic resonance imaging, for cell labelling and separation, drug delivery, and as functional components in magnetically-assisted hyperthermia for cancer treatment. Once iron oxide nanoparticles are functionalized with suitable bioactive substances, the resulting system then performs both the diagnostic and therapeutic actions, giving birth to a novel branch of medicine known as theranostics [9]. Many iron oxide-based (both maghemite or magnetite) nanocarriers designed and fabricated for magnetic drug delivery purposes have been reported in the literature to date, involving various synthetic approaches and the use of different organic-coating layers [5,6,10,11,12,13,14,15,16,17,18,19]. Regarding the nanocomposites with maghemite-based core-shell NPs functionalized with platinum-based drugs (*i.e.*, structurally similar to those reported in this work), to the best of our knowledge only two such works have been reported to date. Both of them report maghemite-based NPs with dechlorinated cisplatin bound to 4-oxo-4-(3-(triethoxysilyl)propylamino)butanoic acid (OTPBA) [20,21]. These systems showed remarkable, time-dependent *in vitro* cytotoxicity against MCF7, HeLa, A549 and A549R human cancer cell lines, which is comparable with that of cisplatin after 72 h, and moreover, they can be simultaneously used as MRI contrast agents. Several other works have dealt with similar magnetite-based NPs suitable for magnetic drug delivery and studied cisplatin as the model drug as follows: Deng and Lei reported the Fe_3_O_4_/SiO_2_ cores with PEG–PLA shell (PEG = polyethylene glycol, PLA = polylactic acid) and loaded cisplatin [22]. A similar system, but with a PLA shell, was studied by Devi *et al.*, who focused on loading and release properties of cisplatin [23]. Ashjari *et al.* published the preparation of cisplatin-functionalized magnetite NPs with biodegradable poly(lactic-co-glycolic acid) (PLGA) with different morphological properties of the resulting composite [24]. Several other studies focused on the analogical systems (magnetite core and cisplatin as the functionalizing agent) differing in organic shells, such as folate acid- [25], squalene- [26], carboxymethylcellulose- [27], dextrane- [28] or poly(ethyl-2-cyanoacrylate)- [29] based layer. Finally, Li *et al.* studied the effect of cisplatin-loaded magnetite-based NPs on multidrug resistance and its mechanism [30]. Although all of the mentioned nanosystems have to be considered as universal in terms of functionalization by the platinum complexes, it has to be noted that to the best of our knowledge only one work [31] has reported iron oxide-based NPs functionalized with platinum complexes other than the mentioned cisplatin. In particular, the magnetite-silica composite nanoparticles were investigated as carriers of a photoactive platinum diimine complex.

**Figure 1:**
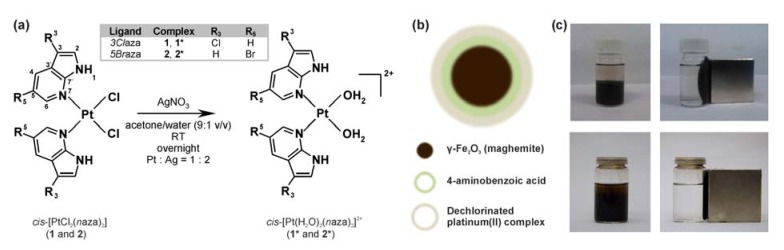
(**a**) The structural formulas of highly cytotoxic *cis*-[PtCl_2_(*3Cl*aza)_2_] (**1**; *3Cl*aza = 3-chloro-7-azaindole) and *cis*-[PtCl_2_(*5Br*aza)_2_] (**2**; *5Br*aza = 5-bromo-7-azaindole), and the corresponding activated diaqua species symbolized as 1* and 2* used for the interaction with PABA@FeNPs; (**b**) the proposed composition of the studied 1*@PABA@FeNPs and 2*@PABA@FeNPs based on the maghemite core coated with 4-aminobenzoic acid and functionalized with 1* or 2*; and (**c**) photos of the obtained aqueous suspensions of PABA@FeNPs (*up*) and 2*@PABA@FeNPs (*down*) without (*left*) and with (*right*) an external magnetic field.

In an effort to research the possibilities of targeted delivery of the recently reported highly *in vitro* cytotoxic cisplatin-like complexes involving 7-azaindole derivatives investigated by our team [32,33,34], we developed a novel system based on easily obtainable magnetic nanoparticles. They consist of maghemite-based 4-aminobenzoic acid (PABA)-coated core-shell nanoparticles (PABA@FeNPs) functionalized by the activated (*i.e.*, dechlorinated) platinum(II) complexes bearing various 7-azaindoles (the complexes **1** and **2**, whose activated form is symbolized as 1* and 2*; Figure 1a). The obtained systems 1*@PABA@FeNPs and 2*@PABA@FeNPs (Figure 1b) were thoroughly characterized by relevant techniques including Mössbauer and FTIR spectroscopy, simultaneous TG/DTA thermal analysis, transmission electron microscopy (TEM) and scanning electron microscopy (SEM) equipped with energy-dispersive X-ray spectroscopy (EDS). The resulting nanocomposites, which were found to be 13.0 ± 2.1 nm of size with acceptable dispersibility, are of high potential from the magnetic drug delivery point of view.

## 2. Results and Discussion

### 2.1. Preparation and Properties of PABA@FeNPs

The maghemite-based nanoparticles (FeNPs) coated with 4-aminobenzoic acid (PABA@FeNPs) were prepared be a method using a mixture of the Fe(III) and Fe(II) salts (FeCl_3_∙6H_2_O and FeCl_2_∙4H_2_O in this work), which was mixed together with PABA in deionized water under atmospheric conditions. NH_4_OH was finally added to the mixture which resulted in the formation of the maghemite-based PABA@FeNPs nanoparticles. It has to be noted that the usual preparation of maghemite-based NPs involves magnetite NPs (prepared from the mixture of Fe(III) and Fe(II) salts under nitrogen [35,36]) and subsequently oxidized by e.g., diluted nitric acid [20,21]. Since we aimed to prepare maghemite-based NPs, we used a different approach—we did not perform the syntheses under nitrogen but under atmospheric conditions, which was found to be sufficient for the magnetite oxidation to occur without addition of any other oxidizing agent. 

**Figure 2:**
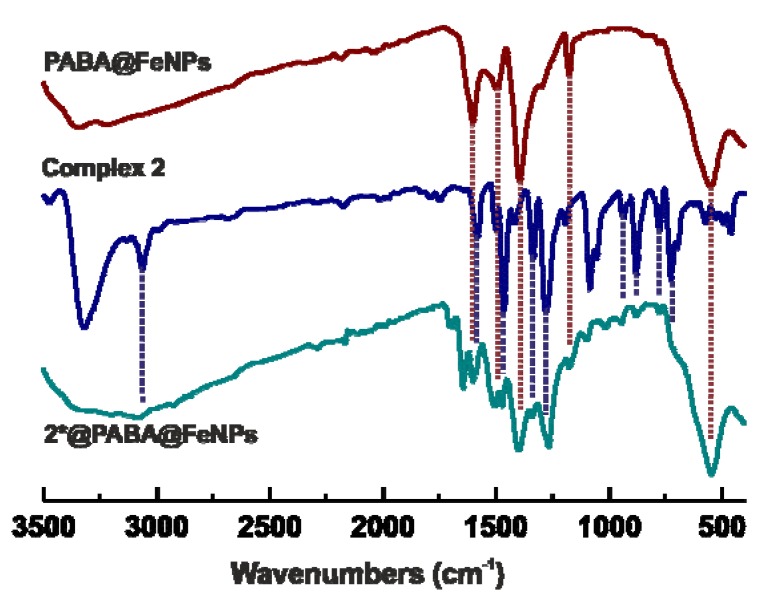
FTIR spectra of the maghemite nanoparticles coated with 4-aminobenzoic acid (PABA@FeNPs; red line), the parent complex **2** involving 5-bromo-7-azaindole (blue line), and the resulting system with the activated complex (2*) bound on the maghemite nanoparticles coated with 4-aminobenzoic acid (2*@PABA@FeNPs).

The presence of the organic layer coating the maghemite NPs within the obtained PABA@FeNPs was proved by the FTIR spectra recorded in the 400–4,000 cm^−1^ region (Figure 2 and Figure S1) as well as by the thermal analysis (Figure S2) [35,37]. The FTIR spectrum of PABA@FeNPs contained the characteristic peak of maghemite at *ca.* 550 cm^−1^ clearly assignable to the n(Fe–O) vibration, as well as a series of peaks, whose positions in the spectrum correlates with those of free PABA molecule (see Figure S1 and Experimental Section). The results of the FTIR spectroscopy also indirectly proved that PABA is bonded to γ-Fe_2_O_3_ within the PABA@FeNPs nanocomposite through the deprotonated carboxyl group, because there is only one major peak in the region characteristic for this functional group with a band centred at 1,603 cm^−1^, as compared with four in total peaks found in the spectrum of free PABA at 1,571, 1,597, 1,623 and 1,660 cm^−1^ (Figure S1).

The PABA@FeNPs hybrid systems exhibited, as expected, a strong attraction to the magnetic field (Figure 1c) and good stability in solution (no macroscopic changes, e.g., colour or magnetism, after one weak of standing in the fridge) and in solid state (usable with no changes of their properties after more than two months of standing in the fridge). 

### 2.2. Functionalization of PABA@FeNPs by Platinum(II) Complexes

We assumed a bonding of the platinum(II) complexes through Pt–N bonds formed between the Pt(II) atom of the activated species and the amino group of PABA. As previously reported, PABA with a substituted carboxyl group (which simulates the bonding of PABA to the maghemite NPs through the mentioned functional group) binds to platinum through the amino group [38,39,40]. Further, the X-ray structures of several platinum(II) complexes involving aniline or its variously substituted derivatives, such as 4-alkylanilines (e.g., [41,42]) are described in the literature. These facts proved PABA is a suitable coating agent for magnetic therapeutic/theranostic systems functionalized with platinum-based agents. 

**Figure 3:**
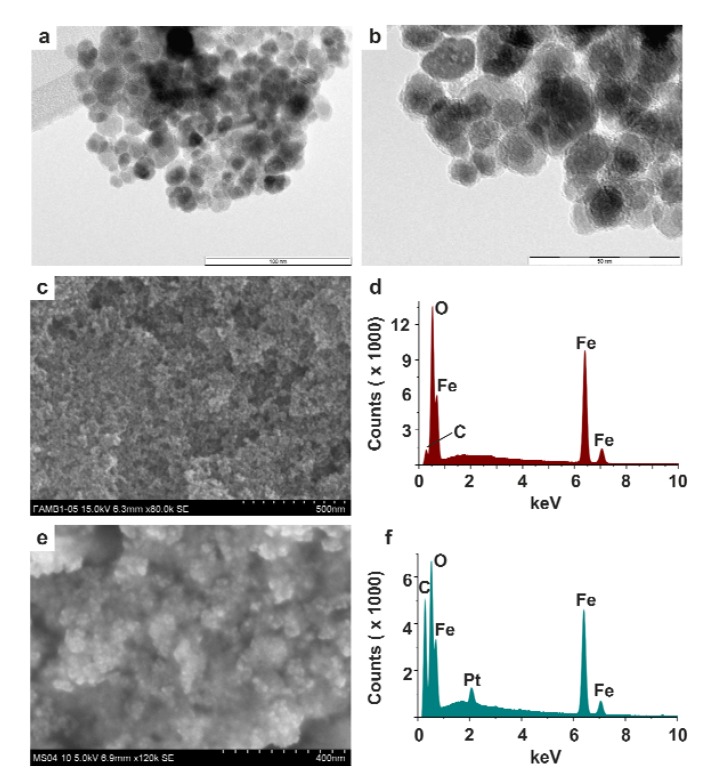
TEM images of 2*@PABA@FeNPs {(**a**) with 100 nm resolution and (**b**) with 50 nm resolution}, SEM images of PABA@FeNPs (**c**) and 2*@PABA@FeNPs (**e**) given with their EDS patterns (**d**, **f**).

The initial *cis*-dichloridoplatinum(II) complexes of the composition *cis*-[PtCl_2_(*n*aza)_2_] (**1** and **2**), which were recently described in the literature by our team as having significant antitumor properties [33,34], were activated by their reactions with a stoichiometric amount of silver(I) nitrate, resulting in dechlorination and formation of the diaquaplatinum(II) species *cis*-[Pt(H_2_O)_2_(*3Cl*aza)_2_]^2+^ (1*) and *cis*-[Pt(H_2_O)_2_(*5Br*aza)_2_]^2+^ (2*). The activated species, involving the labile Pt–aqua bonds which represent suitable sites for consequent formation of the mentioned Pt–N bonds with PABA, were allowed to interact in acetone with the PABA@FeNPs nanoparticles for 48 h. The final systems (1*@PABA@FeNPs and 2*@PABA@FeNPs), which showed strong attraction to the external magnetic field (Figure 1c), were magnetically isolated, purified and stored in the fridge. The presence of the platinum(II) species within the obtained maghemite-based nanocomposite was proved by FTIR spectroscopy (Figure 2) as well as by EDS spectroscopy (Figure 3f). FTIR spectroscopic experiments were performed with the aim to better characterize the studied systems. The detailed analysis of FTIR spectra revealed that bands observed in the spectrum of 1*@PABA@FeNPs at 785, 1,018, 1,099, 1,277, 1,338, 1,516, 1,602, 2,906 and 3,107 cm^−1^ and in the spectrum of 2*@PABA@FeNPs at 700, 883, 946, 1,267, 1,341, 1,473, 1,603 and 3,082 cm^−1^ were not detected in the spectrum of the PABA@FeNPs system. Moreover, the positions of these bands correlate well with those detected in the spectra of the complexes **1** and **2** (Figure 2 for the complex **2** and 2*@PABA@FeNPs), thus showing on the presence of the platinum(II) 7-azaindole species within the resulting nanocomposites. An interpretation of the far-FTIR spectra recorded at 150–600 cm^−1^ provides indirect proof of the covalent bonding between the platinum(II) species and PABA@FeNPs within the studied nanosystems 1*@PABA@FeNPs and 2*@PABA@FeNPs. In particular, the performed far-FTIR experiments regarding **2**, 2* and 2*@PABA@FeNPs indicated changes in inner coordination spheres in the vicinity of the central platinum(II) atom, *i.e.*, the changes going from a PtCl_2_N_2_ donor set (the starting complex 2; two ν(Pt–Cl) maxima at 336 and 345 cm^−1^), through a PtN_2_O_2_ donor set (the dechlorinated complex 2*; two ν(Pt–O) maxima at 322 and 332 cm^−1^) to a PtN_4_ one (the resulting system 2*@PABA@FeNPs; no bands detected in the region mentioned for both the ν(Pt–Cl) or ν(Pt–O) vibrations), as depicted in Figure S3. In other words, although we did not detect the vibrations connected with the anticipated Pt–N bonds between the platinum(II) species and amino group of PABA, we assume that these vibrations are overlapped (analogically to those at *ca* 520 cm^−1^ assignable to Pt–N bonds between the central Pt(II) atom and 7-azaindole rings) by a wide and intensive band belonging to ν(Fe–O). The ν(Fe–O) vibration was, together with a band centred at 579 cm^−1^ assignable to the vibrations connected with the deformation of the 7-azaindole moiety [43], the only band detected in the discussed far-FTIR spectrum of 2*@PABA@FeNPs.TEM images of the prepared 1*@PABA@FeNPs and 2*@PABA@FeNPs systems provided the relevant information regarding the shape, size, and uniformity of the resulting NPs (Figure 3a and Figure 3b). The systems were found to be spherical, core-shell well-dispersed composites with an average size of 13.0 ± 2.1 nm. SEM was used to investigate the surface morphology of the prepared maghemite-based NPs (Figure 3c,e). A comparison of the SEM images depicted for PABA@FeNPs (Figure 3c) and 2*@PABA@FeNPs (Figure 3e) did not show any noticeable difference between their properties, since both systems were detected by SEM (as well as by the above-mentioned TEM) as having a spherical shape of individual NPs, which agglomerated together to the structure without any specific shape. 

Simultaneous TG/DTA thermal analysis revealed a considerable difference between the weight losses of PABA@FeNPs and those involving functionalized platinum(II) complexes (represented by 2*@PABA@FeNPs; Figure S2). PABA@FeNPs did not show any weight increase in the 100–150 °C range (after the loss of water physically adsorbed on the the prepared NPs), which is known to be connected with an oxidation of Fe^2+^ ions, which indirectly proved the chemical composition of the magnetic core (maghemite). The PABA@FeNPs system was thermally stable between 114 and 148 °C, when its thermal degradation (connected with an oxidation of the organic layer coating the maghemite core) started and continued to 460 °C (total weight loss equals 8.0%). The product of the thermal decomposition, most probably γ-Fe_2_O_3_ as previosly reported for maghemite NPs [44], did not show any weight change up to 530 °C, however, an exothermic effect unambiguously assignable to the γ-Fe_2_O_3_ to α-Fe_2_O_3_ conversion was detected on the DTA curve with the maximum at 488 °C (Figure S2). A considerably different weight loss (19.8%) as well as TG-curve shape (a continual decomposition) was found for 2*@PABA@FeNPs indirectly proving the presence of the platinum(II) species within the resulting system (Figure S2).

### 2.3. Mössbauer Spectroscopy

**Figure 4:**
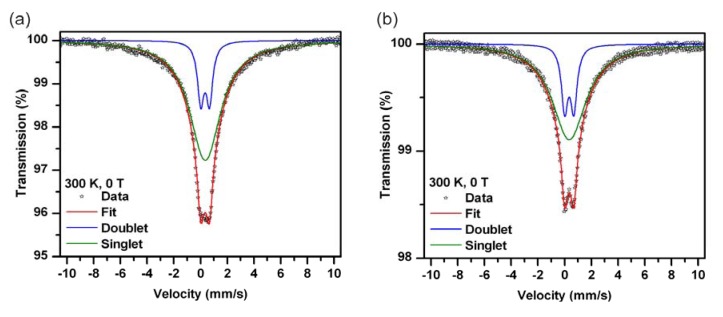
Room-temperature Mössbauer spectra of (**a**) PABA@FeNPs and (**b**) 2*@PABA@FeNPs; doublet - assigned to the Fe^3+^ relaxation component, singlet - asigned to the Fe^3+^ superparamagnetic component.

The ^57^Fe Mössbauer spectra of the samples studied are depicted in Figure 4, while the values of the Mössbauer hyperfine parameters, derived from the fitting of the recorded Mössbauer spectra, are listed in Table 1.

**Table 1:** Values of the Mössbauer hyperfine parameters, derived from the fitting of the room-temperature Mössbauer spectra of PABA@FeNPs and 2*@PABA@FeNPs, where *δ* is the isomer shift, Δ*E_Q_* is the quadrupole splitting and RA is the spectral area of the individual spectral components.

Sample	Component	*δ* ± 0.01 (mm/s)	Δ*E_Q_* ± 0.01 (mm/s)	RA ± 1 (%)	Assignment
PABA@FeNPs	Doublet	0.34	0.66	18	Fe^3+^ relaxation component
	Singlet	0.35		82	Fe^3+^ superparamagnetic component
2*PABA@FeNPs	Doublet	0.35	0.69	88	Fe^3+^ relaxation component
	Singlet	0.35		78	Fe^3+^ superparamagnetic component

The room-temperature Mössbauer spectrum of both the PABA@FeNPs and 2*@PABA@FeNPs sample shows only relaxation components (*i.e.*, singlet and doublet, see Figure 4a and Figure 4b, respectively) with the values of the Mössbauer hyperfine parameters (see Table 1) typical of high-spin Fe(III) atom in iron(III) oxides [45]; there is no indication of presence of the Fe^2+^ valence state. Thus, the nanoparticles in both samples are solely of -Fe_2_O_3_ origin. This is expected in connection with their small size with a large surface area securing their complete oxidation. On the timescale of the Mössbauer technique, all the nanoparticles in both assemblies behave in a superparamagnetic manner at room temperature. The doublet component belongs to the nanoparticles with thermally fluctuating superspins having relaxation times much smaller than the characteristic measurement time (*τ*_m_) of the Mössbauer spectroscopy, while the presence of a singlet corresponds to those nanoparticles the superspin of which thermally fluctuates between the energetically favored orientations with a relaxation time close to *τ*_m_. The PABA@FeNPs and 2*@PABA@FeNPs systems would show superparamagnetic features in the magnetization measurements at room temperature, however, their magnetic characteristics will be significantly driven by finite-size and surface effects (manifested, for example, by a smaller saturation magnetization or lack of saturation and reduced magnetic response under small applied magnetic fields).

## 3. Experimental

### 3.1. Materials and Methods

The starting materials K_2_[PtCl_4_], 3-chloro-7-azaindole (*3Cl*aza), 5-bromo-7-azaindole (*5Br*aza), iron(III) chloride hexahydrate (FeCl_3_∙6H_2_O), iron(II) chloride tetrahydrate (FeCl_2_∙4H_2_O), *p*-aminobenzoic acid (PABA), 25% NH_4_OH, silver nitrate (AgNO_3_) and solvents were supplied by Sigma‑Aldrich Co. (Prague, CzechRepublic) and Acros Organics Co. (Pardubice, CzechRepublic), and used as received. The platinum(II) complexes *cis*-[PtCl_2_(*3Cl*aza)_2_] (**1**) and *cis*-[PtCl_2_(*5Br*aza)_2_] (**2**) (Figure 1a) were prepared as described in our recent paper [33].

Transmission electron microscopy (TEM) was carried out on a JEOL 2010 microscope (200 kV, 1.9 Å point-to-point resolution). A drop of high-purity water with the ultrasonically dispersed samples was placed onto a holey-carbon film supported by a copper-mesh TEM grid and dried in air at room temperature. Scanning electron microscopy (SEM) was performed, together with energy-dispersive X-ray (EDS) spectroscopy, by a Hitachi 6600 FEG microscope (5–15 keV accelerating voltage; the dried samples were placed on an aluminum holder equipped with double-sided adhesive carbon tape). The ^57^Fe Mössbauer spectra of the studied samples were recorded at room temperature employing a Mössbauer spectrometer operating at the constant acceleration mode and equipped with a 50 mCi ^57^Co(Rh) source. The isomer shift values are related to α-Fe at room temperature. The Mössbauer spectra were fitted with the MossWinn software program; prior to fitting, the signal-to-noise ratio was enhanced by a statistically based algorithm [46]. Infrared spectra (400–4000 cm^−1^ and 150–600 cm^−1^ regions) were recorded on a Nexus 670 FT-IR (Thermo Nicolet, Waltham, MA, USA) using the ATR technique. Simultaneous thermogravimetric (TG) and differential thermal (DTA) analyses were performed using an Exstar TG/DTA 6200 thermal analyzer (Seiko Instruments Inc., Chiba, Japan) from room temperature to 650 °C (5.0 °C min^−1^) in dynamic air atmosphere (50 mL min^−1^).

### 3.2. PABA@FeNPs Nanoparticles

4-Aminobenzoic acid (PABA; 0.62 g, 4.5 mmol) was, due to its poor solubility at laboratory temperature, dissolved in hot (80 °C) deionized water (75 mL) and then, FeCl_3_∙6H_2_O (1.17 g; 4.3 mmol) dissolved in deionized water (5 mL) was added. The mixture was stirred at 80 °C for 30 min and then FeCl_2_∙4H_2_O (0.86 g; 4.3 mmol) dissolved in deionized water (2 mL) was added to the solution. After 30 min of stirring at 80 °C the last reagent (10 mL of 25% NH_4_OH) was slowly added. The solution turned dark black as the PABA@FeNPs formed. The suspension was intensively stirred at 80 °C for 60 min. Finally, nanoparticles were magnetically isolated and washed with deionized water (3 × 20 mL) and acetone (3 × 20 mL). Part of the final ferrofluid suspension of PABA@FeNPs (Figure 1c) was stored under degassed acetone in the fridge, while the rest of the product was dried with nitrogen gas and in desiccator over silica gel, and stored in the fridge. PABA@FeNPs: FTIR (*ν*_ATR_/cm^−1^): 552vs, 783w, 1,179m, 1,395vs, 1,493m, 1,603s, 3,219s, 3,334s.

### 3.3. Synthesis of 1*@PABA@FeNPs and 2*@PABA@FeNPs

The complexes **1** (110 mg; 0.2 mmol) and **2** (130 mg; 0.2 mmol) were dissolved in acetone (10 mL) and two molar equivalents of AgNO_3_ dissolved in a minimum volume of deionized water were added. The mixture was stirred at laboratory temperature in the dark for 24 h. After that, AgCl was removed by filtration and washed with acetone (3 × 1 mL) to produce the filtrate containing the solution of the dechlorinated complexes of the composition *cis*-[Pt(H_2_O)_2_(*3Cl*aza)_2_]^2+^ (1*), and *cis*-[Pt(H_2_O)_2_(*5Br*aza)_2_]^2+^ (2*) (Figure 1a). PABA@FeNPs (0.5 mL of the acetone suspension involving 100 mg of PABA@FeNPs) was poured in and the mixture was stirred for 48 h to produce the final systems 1*@PABA@FeNPs and 2*@PABA@FeNPs (Figure 1b,c). These products were magnetically isolated, washed with acetone (3 × 20 mL), dried (nitrogen and then in desiccator over silica gel) and stored in the fridge. 1*@PABA@FeNPs: FTIR (*ν*_ATR_/cm^−1^): 550vs, 785w, 1,018m, 1,099w, 1,179m, 1,208m, 1,277s, 1,338s, 1,398vs, 1,516m, 1,602s, 1,694w, 2,906s, 3,107s. 2*@PABA@FeNPs: FTIR (*ν*_ATR_/cm^−1^): 547vs, 700w, 883w, 946w, 1,017w, 1,101w, 1,177m, 1,267vs, 1,341s, 1,400vs, 1,473s, 1,500s, 1,603m, 1,646s, 1,694w, 3,082s. For far-FTIR spectra of **2**, 2*, PABA@FeNPs and 2*@PABA@FeNPs see Supplementary Materials.

## 4. Conclusions

A simple approach was applied to obtain magnetic 4-aminobenzoic acid-coated maghemite nanoparticles with good stability in solution, with high magnetic response to the external magnetic field and showing superparamagnetic behaviour as proved by the Mössbauer spectroscopy experiments at room temperature. The systems were designed to be able to bind highly cytotoxic platinum(II) complexes involving 7-azaindole derivatives represented by the diaquaplatinum(II) species 1* and 2* prepared by a Ag(I) ion-assisted activation from the initial highly cytotoxic dichlorido complexes. The incorporation of the platinum(II) species was proved by relevant techniques (FTIR, EDS), while a combination of the microscopic techniques (SEM, TEM) showed the obtained core-shell nanocomposites as having the spherical shape and an average size of 13 nm in diameter. Although we are aware of the fact that other important properties of the reported systems (e.g., release of the complex, *in vitro* cytotoxicity, *in vitro* toxicity or MRI experiments), should by also elucidated (the results will be a subject of our forthcoming studies), we have reason to believe that the prepared systems fulfil the basic requirements (magnetism, size, dispersibility or functionalization with therapeutically active substance) for the nanoparticles used in the field of theranostic nanomedicine.

## Supplementary Materials

Supplementary materials can be accessed at: http://www.mdpi.com/19/2/1622/s1.
