# Supplementary material for: 4-Aminobenzoic Acid-Coated Maghemite Nanoparticles as Potential Anticancer Drug Magnetic Carriers: A Case Study on Highly Cytotoxic Cisplatin-Like Complexes Involving 7-Azaindoles
Source: Molecules. 2014 Jan 28;19(2):1622–34. doi: 10.3390/molecules19021622 (PMC6271776; doi:10.3390/molecules19021622)

**Supplementary Materials**

**Figure S1.** FTIR spectra of maghemite nanoparticles (black line), 4-aminobenzoic acid (PABA; dark yellow line) and maghemite nanoparticles coated with 4-aminobenzoic acid (PABA@FeNPs; red line).

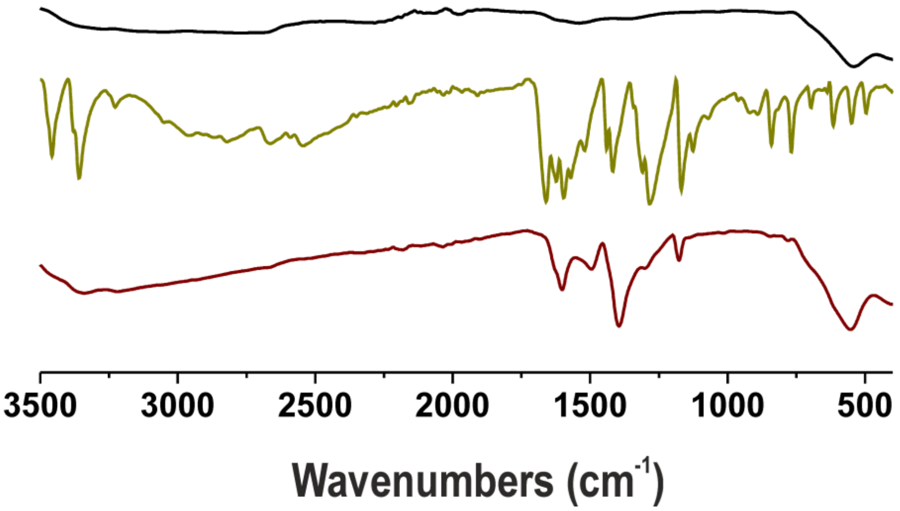


**Figure S2.** The thermogravimetry (TG) results obtained for the PABA@FeNPs (red line) and **2***@PABA@FeNPs (green line) and their comparison with uncoated maghemite nanoparticles (FeNPs; black line), supplemented by the DTA results of PABA@FeNPs (dashed red line).

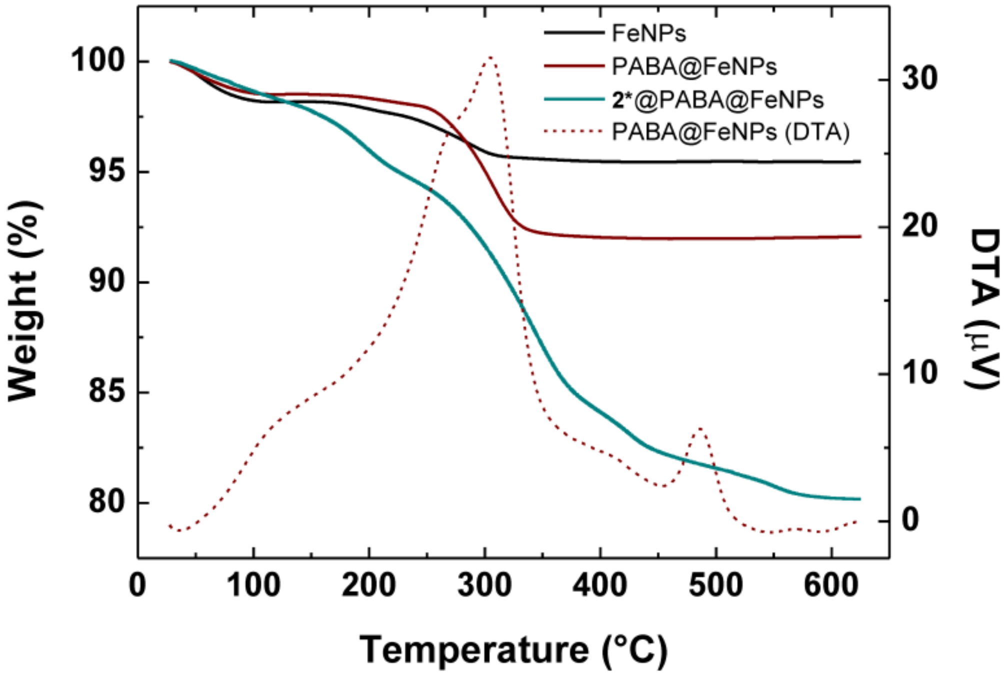

**Figure S3.** The far-FTIR spectra of PABA@FeNPs (red line), the complexes **2** (blue line) and **2*** (dark yellow line), and **2***@PABA@FeNPs (green line) given together with assignment of the ν(Fe–O), ν(Pt–Cl), ν(Pt–O), ν(Pt–$N_{aza}$) and ring deformation vibrations, which indirectly show on the covalent bonding between the platinum(II) species and amino groups of PABA molecules of PABA@FeNPs.

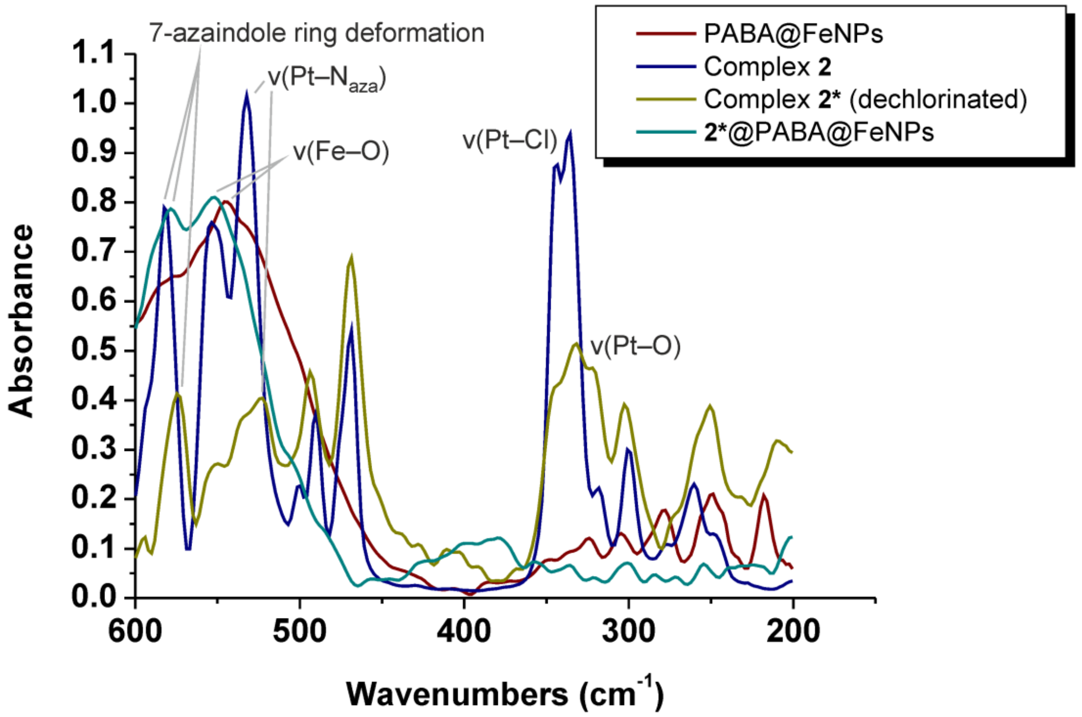

Supplement: Supplementary file 1 [file molecules-19-01622-s001.pdf]
